# ChatGPT in medicine: an overview of its applications, advantages, limitations, future prospects, and ethical considerations

**DOI:** 10.3389/frai.2023.1169595

**Published:** 2023-05-04

**Authors:** Tirth Dave, Sai Anirudh Athaluri, Satyam Singh

**Affiliations:** ^1^Internal Medicine, Bukovinian State Medical University, Chernivtsi, Ukraine; ^2^Rangaraya Medical College, Kakinada, Andhra Pradesh, India; ^3^GSVM Medical College, Kanpur, Uttar Pradesh, India

**Keywords:** ChatGPT, artificial intelligence, AI, natural language processing, generative pre-training transformer, medicine, healthcare

## Abstract

This paper presents an analysis of the advantages, limitations, ethical considerations, future prospects, and practical applications of ChatGPT and artificial intelligence (AI) in the healthcare and medical domains. ChatGPT is an advanced language model that uses deep learning techniques to produce human-like responses to natural language inputs. It is part of the family of generative pre-training transformer (GPT) models developed by OpenAI and is currently one of the largest publicly available language models. ChatGPT is capable of capturing the nuances and intricacies of human language, allowing it to generate appropriate and contextually relevant responses across a broad spectrum of prompts. The potential applications of ChatGPT in the medical field range from identifying potential research topics to assisting professionals in clinical and laboratory diagnosis. Additionally, it can be used to help medical students, doctors, nurses, and all members of the healthcare fraternity to know about updates and new developments in their respective fields. The development of virtual assistants to aid patients in managing their health is another important application of ChatGPT in medicine. Despite its potential applications, the use of ChatGPT and other AI tools in medical writing also poses ethical and legal concerns. These include possible infringement of copyright laws, medico-legal complications, and the need for transparency in AI-generated content. In conclusion, ChatGPT has several potential applications in the medical and healthcare fields. However, these applications come with several limitations and ethical considerations which are presented in detail along with future prospects in medicine and healthcare.

## 1. Introduction

ChatGPT is an advanced language model that leverages deep learning techniques to produce human-like responses to natural language inputs (Radford et al., 2018). It is a member of the family of generative pre-training transformer (GPT) models developed by OpenAI and is currently one of the largest publicly available language models. Using a vast corpus of text data, ChatGPT is capable of capturing the nuances and intricacies of human language, allowing it to generate appropriate and contextually relevant responses across a broad spectrum of prompts (Radford et al., 2018; OpenAI., 2022). Despite the long-standing use of AI (Artificial Intelligence) in various fields such as customer support and data management, its implementation in healthcare and medical research sectors has been relatively restricted. The utilization of AI in healthcare systems is crucial and imperative due to its ability to enhance precision and accuracy while reducing the time required for various aspects of the system. The long-term benefits of incorporating AI in various aspects of healthcare can lead to improved efficiency and accuracy in the sector. The potential applications of ChatGPT in the medical field range from identifying potential research topics to assisting professionals in clinical and laboratory diagnosis (Kharat, 2022). However, these applications come with several limitations and ethical considerations like credibility (van Dis et al., 2023), plagiarism (Biswas, 2023; King and ChatGPT, 2023), and more which are presented in more detail later in this paper. Additionally, recent discussions have emerged regarding the authorship of ChatGPT in research papers, which entail various criteria and considerations that will be explored in this paper. Until now, only a few studies have documented the use and different aspects of ChatGPT in the medical and healthcare fields. This paper provides an analysis and presentation of the advantages, limitations, ethical considerations, future prospects, and practical applications of ChatGPT and AI in the healthcare and medical domains.

## 2. Advantages and applications

**Figure 1:**
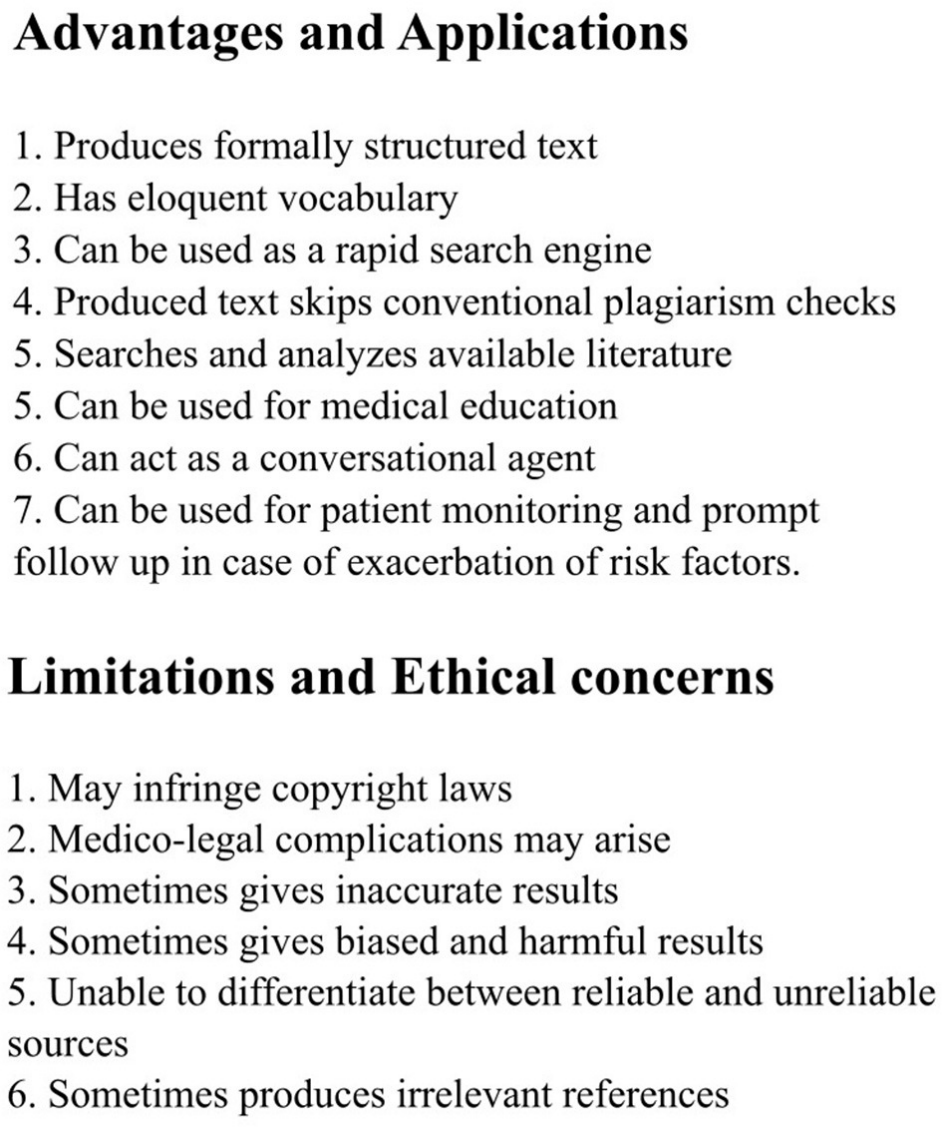
Overview of applications, advantages, limitations, and ethical concerns.

ChatGPT boasts a plethora of applications that can be put to use. Several articles have reported on the use of ChatGPT for writing scientific literature, with one study demonstrating that ChatGPT can produce formal research articles (Gordijn and Have, 2023). The authors discovered that the vocabulary is eloquent, seems to have a conventional tone, and is pleasant to read. ChatGPT can potentially be used as a search engine that replies to queries directly rather than directing to sites where the answers must be obtained by the user—making writing research papers easier and reducing the time spent by the authors in the daunting process of searching for articles and applying various selection criteria to choose the articles best suiting their research, and in turn allowing them to allocate this time on their actual research work and methodology. It can also be used as an intermediary in an ideation session, thus assisting in topic selection and providing a beginning to a research project (Figure 1). Furthermore, ChatGPT-generated articles appear to bypass traditional plagiarism detection methods. In a study, the chatbot was assigned to produce 50 medical research abstracts using a subset of articles published in prestigious journals such as JAMA, The New England Journal of Medicine, The BMJ, The Lancet, and Nature Medicine. The resultant articles were subsequently subjected to scrutiny by plagiarism detection software, an AI-output detector, and a group of medical researchers, who were asked to identify any artificially-generated abstracts. It was found that ChatGPT-generated abstracts smoothed through the plagiarism detection software: the median originality score was 100%, showing that no plagiarism was detected while the AI-output checker identified only 66% of the generated abstracts (Else, 2023).

Moreover, it can also reduce the time, energy, and resources spent on experiments that have a higher probability to obtain futile results by analyzing huge amounts of available literature beyond the scope of a single individual's expertise (Cahan and Treutlein, 2023). It has a “brain” like a person and successfully recalls previous interactions with it as well as prior user comments—a quality that previous AI language models have often been weak at (Chatterjee and Dethlefs, 2023).

The development of virtual assistants to aid patients in managing their health is another important application of ChatGPT in medicine. It can be utilized to generate automated summaries of patient interactions and medical histories, making the medical recordkeeping process more streamlined for doctors and nurses. By dictating their notes, medical professionals can leverage ChatGPT to automatically summarize key details, such as symptoms, diagnoses, and treatments, as well as extract relevant information from patient records, such as lab results or imaging reports. It can also aid in clinical trial recruitment by analyzing large amounts of patient data to identify individuals who meet the trial's eligibility criteria. In addition, ChatGPT can also assist patients in managing their medications by providing reminders, dosage instructions, and information about potential side effects, drug interactions, and other important considerations (Marr, n.d.). According to a recent article on the benefits of artificial intelligence for the self-management of sickle cell disease, ChatGPT can serve as a reliable conversational agent to collect information from patients with a diverse range of diseases (Issom et al., 2021).

It can also be used to help medical students, doctors, nurses, and all members of the healthcare fraternity alike to know about updates and new developments in their respective fields, and can also be used as a tool of assessment to assess clinical skills (Han et al., 2022) thus playing a pivotal role in medical education. Chatbots like ChatGPT can be used as powerful tools to increase health literacy, especially among students and young adults (Mokmin and Ibrahim, 2021).

ChatGPT can potentially also be used for diagnosis. There has been a recent surge in interest in AI-enabled chatbot-based symptom checker (CSC) applications that employ human-like interactions to deliver potential diagnoses, and support users with self-triaging depending on Artificial Intelligence (AI) approach (You and Gui, 2020). Moreover, ChatGPT also can be used in clinical decision support and patient monitoring, suggesting consultation with a healthcare professional based on warning signs and symptoms.

## 3. Limitations

Despite its potential applications, the use of ChatGPT and other artificial intelligence (AI) tools in medical writing also poses ethical and legal concerns. These include possible infringement of copyright laws, medico-legal complications, and the potential for inaccuracies or prejudices in the generated content. Therefore, it is important to acknowledge and address the limitations and issues associated with the use of AI in medical writing. According to two papers from radiology journals, the accuracy of the text generated by AI models is heavily dependent on the quality and nature of the training data used, and in some cases, the output of such models can be incorrect, leading to potential legal issues such as lawsuits. Inaccuracies, biases, and transparency are additional issues that need to be addressed when using AI-generated text. The unethical utilization of AI technology may extend to fabricating images, which constitutes a type of scientific misconduct. Additionally, the data fed into it is only up to 2021 (Kitamura, 2023; Shen et al., 2023). Furthermore, there are several other limitations that hinder its usefulness for research purposes, such as the tendency to provide inaccurate responses and repeat phrases from previous interactions. Additionally, it can be overly sensitive to variations in the phrasing of questions and struggles to clarify ambiguous prompts (Gordijn and Have, 2023). Chatbots can struggle to identify important information and differentiate between reliable and unreliable sources, similar to the biases that humans face. This can limit their usefulness in research, as they may only replicate what has already been done without adding human-like scientific insights. As a result, some scientists oppose using chatbots for research (van Dis et al., 2023).

ChatGPT-generated text can be identified by made-up quotes and irrelevant references, which can help to identify instances of plagiarism and other issues. However, there are still concerns about the potential for students and scientists to use ChatGPT to deceive others or pass off its generated text as their own (Graham, 2022). It should be emphasized that, at present, language models like ChatGPT are not capable of completely taking over the role of human writers, as they lack a similar level of comprehension and specialized knowledge in the field of medicine. Thus, those who employ language models in their use must acknowledge these limitations and take measures to guarantee the precision and reliability of the written materials (Biswas, 2023).

## 4. Ethical considerations

ChatGPT adheres to the EU's AI ethical guidelines, which emphasize the crucial role of human oversight, technical robustness and safety, privacy and data governance, transparency, diversity and non-discrimination, societal and environmental well-being, and accountability in the development and deployment of AI systems. These guidelines prioritize the importance of empowering humans, ensuring safety and accuracy, respecting privacy, preventing unfair bias, promoting sustainability, and providing accountability mechanisms and redress options for negative outcomes (Ethics guidelines for trustworthy AI | Shaping Europe's digital future., 2019). Most articles in scientific literature seem to focus only on the ethical concerns pertaining to the use of ChatGPT in medical literature, as its other applications in medicine are yet to be put into widespread use. The incorporation of AI in literature raises questions regarding authorship and accountability for the produced content. Although ChatGPT creates articles with less plagiarism, they are not totally free of it and require human editing. Letters of recommendation and personal remarks based on works made by chat GPT might also raise doubts about legitimacy (Biswas, 2023). When AI-generated language is used for commercial reasons, it is crucial to make sure that it does not violate any underlying copyrights. ChatGPT, according to publishers and preprint servers contacted by Nature's news team, does not meet the criteria for a study author since it cannot bear responsibility for the content and authenticity of scientific studies (Stokel-Walker, 2023).

ChatGPT might serve the social purpose of removing language barriers and by extension allowing more individuals to produce high-quality medical literature. However, as with other technologies, high-income nations and privileged academics are likely to eventually discover methods to leverage LLMs in ways that advance their research while exacerbating disparities. As a result, discourses must involve individuals from under-represented groups in research as well as people from communities affected by the research work in order to utilize people's personal experiences as a valuable resource. Authorship definitions must be examined and specified (van Dis et al., 2023).

As algorithms like ChatGPT become more sophisticated and efficient in the coming years because they are updated with ever-increasing data from the internet and beyond, there is a strong likelihood that they will be misused and manipulated. One study notes that some users attempted to reword queries and ask the model how to shoplift without imposing moral restrictions; the model opted to cooperate and supplied extensive information on shoplifting strategies (Chatterjee and Dethlefs, 2023). Hence, it is important that individuals use it as a supplementary resource instead of solely relying on it, and verify the content generated by it keeping ethical considerations in mind.

## 5. Future prospects

In the future, ChatGPT will be widely used and integrated into all text editing programs (Kitamura, 2023). Advanced systems should be developed which can focus on identifying even the small manipulation in the data done by the ChatGPT (Shen et al., 2023). Currently, identifying the output of ChatGPT requires meticulous inspection by editors, and its inability to properly cite resources poses a challenge. However, ongoing research aims to address these issues and propose effective solutions (Editorials, 2023). Strict guidelines should be implemented by the journals regarding AI use in scholarly papers to minimize its misuse (Liebrenz et al., 2023). Instead of solely relying on detecting the limitations of ChatGPT for detecting potential academic misconduct, educators can alter assignment questions to prioritize critical thinking skills, thus limiting opportunities for improper use of technology (Graham, 2022). ChatGPT, while not connected to the internet and having limited knowledge, may produce inaccurate or biased content. However, users can provide feedback using the “Thumbs Down” button, which enables ChatGPT to learn and improve its responses. Additionally, responses generated by ChatGPT are reviewed by AI trainers to improve the system's performance. The collected data is securely stored to ensure user privacy and may be deleted upon request (ChatGPT General FAQ, n.d.).

## 6. Discussion

ChatGPT is a tool that can assist in various domains of healthcare and medicine such as in structuring scientific literature, analyzing vast literature, and functioning as a conversationalist agent. It has many advantages and applications in the system but they cannot be enforced without knowing and understanding its limitations and potential ethical concerns arising from its use like infringing copyright laws, biases, and accountability dilemmas.

ChatGPT can significantly reduce the time required to perform various tasks. However, its use in tasks such as summarizing patient data, research tasks, and AI-enabled CSC has been limited to a small number of individuals. If used wisely, the time saved by using ChatGPT can be utilized for more productive and prioritized tasks. It can also be used by healthcare professionals to translate and explain patient medical notes or diagnoses in a more patient-friendly way (Implications of ChatGPT for Healthcare, n.d.).

Overcoming the current limitations of ChatGPT, such as improving and training the chatbot to provide 100% accurate and unbiased information from the sources, is essential to address the accountability concerns that have prevented the majority of journals from allowing ChatGPT as an author. However, some preprint servers are allowing submitting ChatGPT as an author along with human authors. To address the accountability dilemma, one possible solution is for authors to include a clear explanation in the methods or other relevant sections of their paper about how they used ChatGPT as a tool to assist them in their research. This can help the journal and readers to acknowledge the role of ChatGPT in the research process and ensure transparency. Developing appropriate protocols and establishing clear lines of responsibility can help mitigate the risks associated with the use of ChatGPT in research and writing (Editorials, 2023). If used with proper supervision ChatGPT can assist all from medical students to doctors in their professional tasks and help them work efficiently.

## 7. Conclusion

ChatGPT is a state-of-the-art language model that has numerous advantages and applications in the healthcare and medical domains. It can assist medical professionals in various tasks, such as research, diagnosis, patient monitoring, and medical education. However, the use of ChatGPT also presents several ethical considerations and limitations such as credibility, plagiarism, copyright infringement, and biases. Therefore, before implementing ChatGPT, the potential limitations and ethical considerations need to be thoroughly assessed and addressed. Future research can focus on developing methods to mitigate these limitations while harnessing the benefits of ChatGPT in the healthcare and medical sectors.

## Author contributions

TD and SA: conception and design of the paper and literature search. TD, SA, and SS: literature review and data collection. All authors contributed to the writing, revising, and submission of the final draft.
